# Clinical outcomes and neural correlates of 20 sessions of repetitive transcranial magnetic stimulation in severe and enduring anorexia nervosa (the TIARA study): study protocol for a randomised controlled feasibility trial

**DOI:** 10.1186/s13063-015-1069-3

**Published:** 2015-12-03

**Authors:** Savani Bartholdy, Jessica McClelland, Maria Kekic, Owen G. O’Daly, Iain C. Campbell, Jessica Werthmann, Samantha J. Rennalls, Katya Rubia, Anthony S. David, Danielle Glennon, Nikola Kern, Ulrike Schmidt

**Affiliations:** Section of Eating Disorders, Department of Psychological Medicine, Institute of Psychiatry, Psychology and Neuroscience, King’s College London, London, UK; Centre for Neuroimaging Sciences, Department of Neuroimaging, Institute of Psychiatry, Psychology and Neuroscience, King’s College London, London, UK; Department of Child and Adolescent Psychiatry, Institute of Psychiatry, Psychology and Neuroscience, King’s College London, London, UK; Department of Psychosis Studies, Institute of Psychiatry, Psychology and Neuroscience, King’s College London, London, UK; South London and Maudsley NHS Foundation Trust, London, UK

**Keywords:** Anorexia nervosa, Treatment, Trial, Transcranial magnetic stimulation, Neuromodulation, Neuroimaging

## Abstract

**Background:**

Anorexia nervosa (AN) is a serious mental disorder with multiple comorbidities and complications. In those with a severe and enduring form of the illness (SEED-AN), treatment responsivity is poor and the evidence base limited. Thus, there is a need for novel treatment strategies. This paper describes the theoretical background and protocol of a feasibility randomised controlled trial (RCT) of real versus sham (placebo) therapeutic repetitive transcranial magnetic stimulation (rTMS) in SEED-AN. The aim of this trial is to obtain information that will guide decision making and protocol development in relation to a future large-scale RCT of rTMS in this group of patients, and also to assess the preliminary efficacy and neural correlates of rTMS treatment.

**Design:**

Forty-four adults from the community with a DSM-5 diagnosis of AN, an illness duration >3 years and a previous course of unsuccessful treatment will be randomly allocated to receive 20 sessions of either real or sham rTMS, in a parallel group design. As this is a feasibility study, no primary outcome has been defined and a broad range of outcome variables will be examined. These include weight/body mass index (BMI), eating disorder psychopathology, other psychopathology (for example, depression, anxiety), quality of life, neuropsychological processes (such as self-regulation, attentional bias and food choice behaviour), neuroimaging measures (that is, changes in brain structure or function), tolerability and acceptability of rTMS, and additional service utilisation.

The feasibility of conducting a large-scale RCT of rTMS and the appropriateness of rTMS as a treatment for SEED-AN will be evaluated through: assessment of recruitment and retention rates, acceptability of random allocation, blinding success (allocation concealment), completion of treatment sessions and research assessments (baseline, post-treatment and follow-up assessments). The acceptability and tolerability of the treatment will be assessed via semi-structured interviews.

**Discussion:**

The effect sizes generated and other findings from this trial will inform a future large-scale RCT with respect to decisions on primary outcome measures and other aspects of protocol development. Additionally, results from this study will provide a preliminary indication of the efficacy of rTMS treatment for AN, the neural correlates of the illness, and potential biomarkers of clinical response.

**Trial registration:**

ISRCTN14329415. Date of registration: 23 July 2015.

**Electronic supplementary material:**

The online version of this article (doi:10.1186/s13063-015-1069-3) contains supplementary material, which is available to authorized users.

## Background

Anorexia nervosa (AN) is a disabling, deadly disorder with a high disease burden [1–4]. Onset is usually peri-pubertal and AN mainly affects females. The lifetime prevalence of AN in women is 2–4 % [5], the median duration is 5–7 years and 30 % have an illness duration greater than 15 years [6]. AN is associated with functional and structural brain changes and some studies find that these correlate with chronicity [7–9]. Converging evidence suggests that after 3–5 years at low weight, treatment responsivity of AN lessens and outcomes become poorer, possibly due to neurotoxic effects of starvation and stress hormones (cortisol) on the brain [10, 11]. Thus, the first years after onset seem critical for successful intervention [12]. For example, 60–70 % of adolescents with AN who typically have a short illness duration (that is, <3 years) make a full recovery with family-based psychological treatments [13, 14], whereas only about 10–30 % adults, who usually have a more enduring form of AN, achieve full remission after a course of the best available psychotherapy [15–18]. Pharmacological treatments (antidepressants, antipsychotics) are largely ineffective and have low acceptability [19–22].

Cases of AN where there is a sustained (>3 years) period of being significantly underweight (body mass index (BMI) < 18.5 kg/m^2^), can be defined as a severe and enduring eating disorder 1 (SEED) [23–25]. For this group, treatment options are limited, especially as these patients will often have had several previous unsuccessful interventions [25]. A systematic review of treatments for severe and enduring anorexia nervosa (SEED-AN [25]) identified only one randomised controlled trial (RCT) (*N* = 63) comparing two psychological therapies, and reported limited improvements in both groups [23]. One further trial [26] evaluated dronabinol (a cannabinoid agonist) against placebo in patients with SEED-AN (*n* = 25) and found a small but significant weight gain. However, only 18% of those approached agreed to participate. Finally, deep brain stimulation (DBS) has shown promise in highly selected SEED-AN patients [27]. As DBS is an invasive procedure, it may not be an appropriate and/or desirable treatment option for the majority of patients with SEED-AN. Thus, there is a strong need for new, non-invasive treatment strategies for this group [28]. Treatment advances are most likely to arise from neuroscience-based interventions that probe and target disease mechanisms [28, 29].

Functional neuroimaging studies of AN using symptom provocation paradigms have proposed aberrant functioning of ‘top-down’ (evaluative) prefrontal regions (involved in executive control) and/or subcortical regions promoting ‘bottom-up’ (stimulus-driven) responses, including anxiety-related mesolimbic circuits, reward-related regions (for example, the striatum) and parietal somatosensory regions [30, 31]. Such findings have been incorporated into disease models that suggest that in AN, altered neural circuitry underlies phenotypic difficulties in the regulation of appetite, emotion and self-control [32–34] and the fact that AN symptoms (such as food restriction and excessive exercise) become rewarding, compulsive and/or habitual with increasing illness duration [35, 36]. This may explain the persistent nature of the illness [37]. Underpinning the hypothesis that altered self-regulation plays a key role in the development and/or maintenance of AN, a number of behavioural and neuroimaging studies have reported that individuals with AN show differences from other eating disorders (EDs) and healthy individuals on tasks of motivational self-control (see, for example, [38]) and response inhibition (see, for example, [39]). Neuroimaging research using such tasks has implicated the dorsolateral prefrontal cortex (DLPFC) in these cognitive processes [40–42]. Such neural models of AN and the role of the DLPFC in self-regulation provide a strong rationale for targeting the DLPFC in treatment.

Neuromodulation techniques, such as repetitive transcranial magnetic stimulation (rTMS), that directly alter brain activity provide a promising avenue for delivering precise brain-directed treatments for mental disorders, such as EDs [27, 28, 43–46]. rTMS is a non-invasive procedure that can be used to stimulate (high frequency) or inhibit (low frequency) cortical activity, and the DLPFC is a widely used target. It has shown promising therapeutic effects in several psychiatric disorders [47–51], leading to its approval by the Food and Drug Administration (FDA) as a second line treatment for depression in the USA. It appears to increase neuroplasticity, that is, the ability of the brain to form new nerve connections, and hence rTMS is proposed to be of particular value in chronic or treatment-resistant neuro-circuit-based disorders [52], such as SEED-AN.

Evidence is accumulating to support rTMS as safe, well-tolerated, and of some benefit in the treatment of EDs. Two recent reviews have summarised the effects of neuromodulatory interventions, including rTMS, on ED symptoms, body weight and food intake in human and animal studies, and highlight the potential for treating EDs [45, 46]. Proof-of-concept studies in EDs have shown that in the short term, high-frequency rTMS applied to the left DLPFC reduces key symptoms [46, 53–55]. A single-session sham-controlled RCT of rTMS indicated that cue-elicited food craving can be reduced in healthy women who have high levels of food craving [56]. In bulimia nervosa, a single session of rTMS has been reported to reduce food craving, binge eating and salivary cortisol levels, suggesting the effects of rTMS on food craving involve the hypothalamic-pituitary-adrenal (HPA) axis [53]. Evidence also suggests key symptoms of AN (for example, urge to restrict food intake, feeling full/fat) are reduced after a single session of rTMS to the left DLPFC [57], McClelland J, Kekic M, Bozhilova N, Nestler S, Dew T, Van Den Eynde F, David A, Rubia K, Campbell IC, Schmidt U, unpublished observations, which appears to occur alongside an improvement in motivational self-control (that is, temporal foresight) [McClelland et al., unpublished observations]. A recent case series of therapeutic rTMS (20 sessions) found sustained improvements (for at least 6 months) in ED symptomatology and/or affective symptoms in five patients with SEED-AN who had had multiple unsuccessful previous treatments [58]. Finally, studies in healthy participants have shown that rTMS to the DLPFC can improve performance on self-regulation tasks, and thus it may have promise in targeting the self-regulation difficulties in EDs [59]. Given the success of rTMS to the DLPFC in treating other neurocircuit-based disorders (such as treatment-resistant depression) and the encouraging preliminary data on its effects on symptoms of AN [46, 58], McClelland et al., unpublished observations, there is a strong rationale for further exploring the therapeutic potential of rTMS in SEED-AN.

In summary, the evidence base for treatment of SEED-AN is limited [23, 25], and use of rTMS appears promising. To date, no sham-controlled RCT of therapeutic rTMS in AN has been conducted. The proposed feasibility study is an RCT comparing real (active) to sham (placebo) high-frequency rTMS to the left DLPFC as an adjunct to treatment as usual (TAU) in individuals with SEED-AN. Participants will be randomly allocated to receive 20 sessions of either real or sham rTMS. Participants’ ED symptoms and other clinical outcomes will be measured before, during, immediately after and 3 months after treatment. Additionally, the effects of rTMS on neuropsychological processes such as self-regulation, attentional bias and food choice behaviour will be explored. Neuroimaging measures will be used pre- and post-treatment to explore neural mechanisms underlying treatment effects. Lastly, participants will be interviewed about their experiences of this treatment.

### Aims

The specific objectives of the proposed feasibility study are to:

1. establish the feasibility of conducting a large-scale RCT of rTMS in patients with SEED-AN by assessing recruitment, attendance and retention rates;
2. determine the best instruments for measuring outcomes in a full trial by examining the quality, completeness and variability in the data;
3. estimate the treatment effect sizes and standard deviations for outcome measures to inform the sample size calculation for a large-scale RCT;
4. evaluate whether the treatment is operating as designed by analysing process measures, for example, within-session visual analogue scales (VAS) of key ED symptoms;
5. explore patients’ views on the acceptability, credibility, tolerability and experience of rTMS;
6. obtain information about patients’ willingness to undergo random allocation to 20 sessions of real or sham rTMS;
7. investigate neurocognitive changes underlying treatment response; and
8. investigate neural mechanisms underlying treatment response, including changes in structure, cerebral blood flow and in regional activity and functional connectivity at rest and during computer-based tasks assessing behavioural control.

Tentative underpinning hypotheses are based on pilot studies previously conducted by our group and others in patients with EDs [53, 55, 58, McClelland et al., unpublished observations] and other disorders that are frequently comorbid with EDs, including depression [60–62] and addictions [63]. It is predicted that, compared to sham rTMS treatment, 20 sessions of high-frequency rTMS applied to the left DLPFC will:

1. reduce AN symptomatology, encourage an increase in weight/BMI and improve related psychopathology (such as depression, anxiety and stress) and quality of life;
2. improve self-regulatory control, emotion regulation, attentional-bias processes, and food choice behaviour. Improvements in self-regulatory control abilities will be associated with changes in task-based neural activity and connectivity between the DLPFC and the inhibitory control network (the right inferior frontal gyrus, pre-supplementary motor area) and areas implicated in reward processing (for example, the ventral striatum [64]);
3. alter neural activity in the DLPFC at rest (for example, cerebral blood flow) and during task performance during functional magnetic resonance imaging (fMRI), which will be correlated with symptom improvement; and
4. be considered by patients as an acceptable and useful treatment adjunct for AN.

## Methods

### Design

**Fig. 1:**
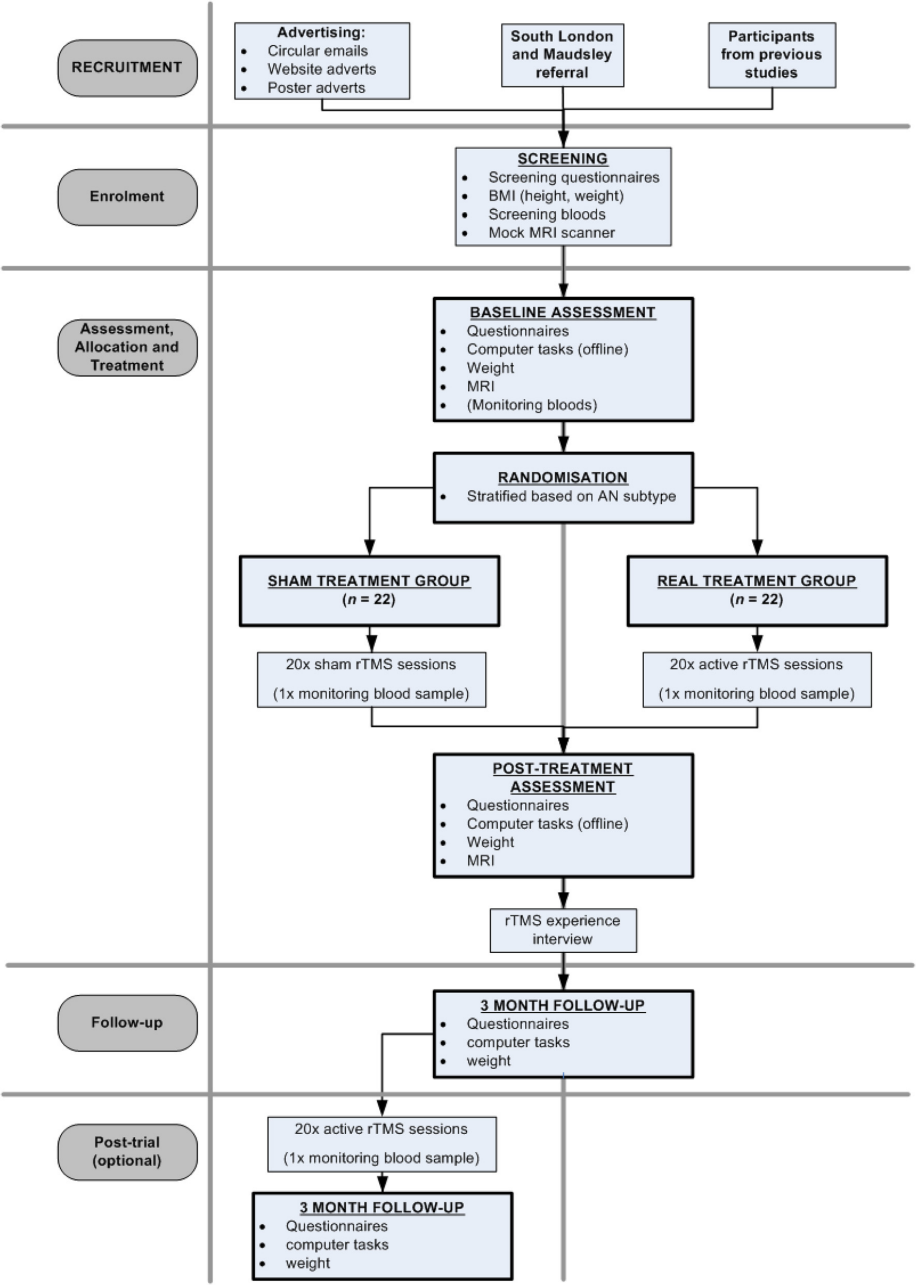
Schematic diagram of the TIARA study protocol

This is a parallel group, double-blind, two-arm RCT. Participants with SEED-AN will be randomly allocated to receive 20 sessions of either real rTMS (treatment group) or sham rTMS (control group) on consecutive weekdays. Participants will be recruited from the community and rTMS will be delivered in addition to TAU. Outcomes will be measured at baseline, post-treatment and 3-month follow-up. Selected clinical outcomes assessing ED symptomatology will additionally be measured during treatment. Participants in the control group will be offered the opportunity to receive real rTMS after the 3-month follow-up. The protocol is outlined in Fig. 1, and Additional file 1 gives details of all assessments and time points.

### Setting

The study will be conducted at the Institute of Psychiatry, Psychology and Neuroscience (IoPPN) in a designated rTMS suite.

### Ethical approval and trial registration

Ethical approval for the TIARA trial was obtained from NRES Committee London - City Road & Hampstead (REC ref: 15/LO/0196). The study is registered on the International Standard Randomised Controlled Trial Number (ISRCTN) registry (registration number: ISRCTN14329415).

### Participants and recruitment

Participants will be recruited from the Eating Disorders Unit in the South London and Maudsley NHS Foundation Trust, through websites (such as IoPPN and Beat [the UK’s leading EDs charity]), and through participation in our previous single session RCT (REC ref: 12/LO/1525) or the Charlotte's Helix/BRC BioResource project (REC ref: 09/H0606/84).

#### Inclusion criteria

Male or female participants will be included if they are right-handed, have a current DSM-5 diagnosis of AN, are aged 18 or over and meet the following criteria for SEED-AN: (1) an illness duration of 3 years or more (this is the minimum illness duration cited in the literature after which treatment outcomes tend to be poorer [11, 25]); (2) a BMI between 14 and 18.5 kg/m^2^ (the upper BMI cut-off of 18.5 kg/m^2^ was chosen pragmatically for comparability with other studies, for example, [23] and the lower BMI limit of 14 kg/m^2^ was selected as a safety precaution); and (3) at least one previous adequate course of ED treatment (for example, one 6-month course of specialist outpatient psychotherapy, specialist day care or in-patient treatment for refeeding). All participants must have agreement from their ED clinician or their GP to take part.

#### Exclusion criteria

Exclusion criteria mainly relate to patients’ safety. Participants will be excluded if they: have a BMI below 14 kg/m^2^; are currently receiving inpatient treatment; are deemed medically (for example, they have significant electrolyte abnormalities) or psychiatrically (for example, they exhibit acute suicidality) unstable by their clinician; require immediate inpatient treatment (for example, due to very low weight and rapid weight loss); have a history of epileptic seizures or head injury; have comorbid major other psychiatric disorders needing treatment in their own right; are on a dose of psychotropic medication that has not been stable for at least 14 days; or have metallic implants anywhere in the body.

#### Sample size

As this is a feasibility study, no a priori sample calculation has been conducted. This study aims to provide effect sizes on which future large-scale studies can be powered. Total sample sizes of *N* = 24–50 have been recommended for feasibility trials with outcomes measured on a continuous scale, mainly because estimates of the standard deviation for normally distributed variables tend to stabilise around this size [65, 66]. We have chosen a sample size of *N* = 40 (that is, at the upper end recommended for feasibility trials). Assuming the attrition to follow-up rate is a = 0.10 (as found in our previous AN trials [15, 67], McClelland et al., unpublished observations and applying an attrition correction factor of 1/(1-a), we will need a total sample size of *N* = 44 (22 participants per group).

### Randomisation

Generation and implementation of the randomisation sequence will be conducted independently from the trial team by the King’s Clinical Trials Unit (CTU). Once the baseline assessment has been carried out and the patient is recruited and has consented to the trial, the researcher will enter patient ID and stratification details into the web-based CTU system. Patients will then be allocated to one of the two trial arms using a restricted stratified randomisation algorithm, stratifying by prognostic factors (type of AN - binge-purge or restricting; intensity of TAU - low (outpatient) or high (daypatient)). The stratification will be implemented by minimised randomisation with a random component. The first n cases (n will not be disclosed) will be allocated entirely at random to further enhance allocation concealment.

### Intervention

#### Commonalities between both groups

In both groups participants will receive 20 real or sham rTMS sessions over 20 consecutive weekdays. Testing/experimental sessions will last between 40–60 minutes, including preparation time, 20 minutes of rTMS and questionnaire administration. Throughout the study, participants will be able to access or continue TAU as recommended by their treating team. TAU will range from GP care only to specialist eating disorder care (day-care or outpatient only).

#### Preparation for rTMS sessions

##### Localisation of the DLPFC

Both groups of participants will undergo a structural magnetic resonance imaging (MRI) scan to locate the left DLPFC (using Brainsight™ neuronavigation software). The protocol includes a high-resolution sagittal 3D T1-weighted volume (voxel size 1.1 × 1.1 × 1.2 mm^3^) based on the well-validated ADNI protocol (http://adni.loni.usc.edu/methods/documents/mri-protocols/).

##### Determining the intensity of the rTMS stimulation

Through mapping of the first dorsal interosseous (FDI) muscle, the intensity of the rTMS will be acquired by obtaining the individual’s motor threshold (MT), which represents membrane-related excitability of cortical axons. To ensure safety and efficacy, the MT will be assessed weekly for each participant during participants’ 20 rTMS treatment sessions. Using the motor evoked potential method (MEPM), the MT is established by determining the minimum stimulator output intensity required to obtain five out of ten motor evoked potentials (MEPs) greater than 50 μV.

#### TMS sessions

A Magstim Rapid^2^ device (Magstim®, UK) and Magstim D70-mm air-cooled real/sham coil will be used to administer real and sham rTMS. Before each rTMS delivery, participants in both groups will watch a short film showing highly palatable foods, which will function as cue exposure to disorder-related stimuli.

##### Delivery of real rTMS

Patients in this group will receive 20 sessions of high-frequency rTMS (10 Hz) at 110 % of their individual MT, consisting of twenty 5-second trains with 55-second inter-train intervals delivered to the left DLPFC [Talairach co-ordinates x = −45 y =45 z = 30 [58, 68].

##### Delivery of sham rTMS

Sham stimulation will be given at the same parameters as the real rTMS; however, a sham coil will be used. The sham coil makes the same noise as the real coil but does not produce a magnetic field.

##### Therapist training and supervision, safety monitoring, side effects, untoward events and study withdrawal

All study procedures and parameters are in accordance with the current safety and application guidelines for rTMS [69]. Treatment will be delivered by personnel trained in the administration of rTMS. A case record form for each trial patient will be kept to monitor session attendance and any side effects or adverse events according to pre-specified criteria. Any protocol violations will also be recorded here. In the event of mild side effects (such as a slight headache) patients will not be withdrawn, but will be able to discontinue rTMS treatment if they wish. rTMS will be immediately halted if the participant experiences a more serious adverse event (such as an epileptic seizure), if their BMI falls below 14 kg/m^2^ or if any other indicators of serious medical risk emerge. Treatment will only be restarted if it is deemed safe to continue by a medical professional.

To ensure safety, patients’ weight, blood pressure (sitting and standing) and pulse will be monitored weekly. Routine blood tests including full blood count, liver and renal function tests will be taken at the start of treatment and repeated at least once during treatment (at approximately session 8) or more frequently if clinically indicated.

### Procedure

A flowchart outlining the study procedures is presented in Fig. 1. For further information about the time schedule of enrolment, interventions and study assessments, please see Additional file 1.

#### Screening

Potential participants will be referred to the study by their clinician or if they self-refer will have to provide their ED team’s or GP’s agreement. Study researchers will screen participants for eligibility. Screening questionnaires include the Eating Disorder Diagnostic Scale (EDDS), the Structured Clinical Interview for DSM Disorders (SCID) screening module, the TMS Adult Safety Screen, an MRI safety screen questionnaire developed at King’s College London and a short inclusion/exclusion screen specific to this study, including an assessment of medical and psychiatric history, and medication dosage and stability. Once eligibility has been confirmed, the patient’s written informed consent will be obtained. Thereafter, participants will be offered a session in a mock MRI scanner to familiarise them with the MRI environment. In line with the CONSORT guidelines [70, 71], we will record the number and reasons for any participants we must exclude, or any who decline consent or withdraw from the study.

#### Baseline assessment

Eligible participants will be weighed and asked to complete a battery of questionnaires relating to ED symptomatology and mood, as well as neuropsychological computer tasks that assess behavioural control, attentional bias and food choice behaviour. In addition a one-hour MRI scan will be conducted. The scan will include structural MRI, fMRI (including the Stop Signal Task (SST) and temporal discounting (TD) task), resting state fMRI and arterial spin labelling (ASL).

Once the baseline assessment is complete, participants will be randomly allocated to the treatment (real rTMS) or control (sham rTMS) group.

#### Post-treatment assessment

The post-treatment assessment will take place in the week following the last rTMS session, and will include the same elements as the baseline assessment.

#### Follow-up

Three months after the post-treatment assessment a follow-up session will be conducted. This session will repeat baseline and post-treatment assessments, except that no MRI scan will be conducted. A qualitative semi-structured interview will also be undertaken with participants (minimum of n = 10 from each real/sham group) to ascertain their views on and experience of this treatment. Finally, blinding success will be evaluated by asking participants and researchers to guess the treatment allocation. Participants will be unblinded and individuals in the sham rTMS group will then be offered the real rTMS treatment.

### Measures

#### Screening measures

- BMI (kg/m^2^)
- Screening questionnaires:
  1. Eating Disorder Diagnostic Scale (EDDS) [72]: This will be used to confirm ED diagnosis. A revised version of the original EDDS incorporating the diagnostic changes in the DSM-5 will be used, although this has not yet been validated [73].
  2. Screening module of the Structured Clinical Interview for DSM Disorders – Researcher Version (SCID) [74]: This short semi-structured psychiatric interview will be used as a diagnostic screen to assess the presence of psychiatric comorbidities.
  3. MRI Safety Screen: This was created at the Centre for Neuroimaging Sciences at King’s College London and will be used to ensure the participant is safe to undergo an MRI scan.
  4. TMS Adult Safety Screen [75]: This will be conducted to check for contraindications to rTMS.
- Other safety assessments: physical observations: blood pressure (sitting and standing), pulse, and routine blood tests.

#### Within-session measures

In all rTMS sessions, participants will complete several VASs before and after rTMS. These will assess perceived levels of stress, anxiety, feeling full, feeling fat, urge to restrict, urge to exercise, urge to binge, urge to purge and mood. At the end of each week, ED and mood symptomatology will be assessed using three short questionnaires: the Eating Disorder Examination Questionnaire short version (EDE-QS) [76], the Fear of Food Measure (FoFM) [77] and the 21 item Depression, Anxiety and Stress Scales (DASS-21) [78].

#### Outcome measures

Since this is a feasibility study, a broad range of outcome measures are included to help determine which are most sensitive to detecting a treatment effect. This will enable us to determine primary outcome(s) for a future large-scale RCT. However, the Eating Disorder Examination Questionnaire (EDE-Q) and DASS-21 scores are the most likely candidates (based on our pilot data) [58].

#### Clinical outcomes

1. ED-related measures: BMI and ED symptomatology (measured by the EDE-Q version 6.0 [79], FoFM, and the Self-Starvation Scale (SS) [80] will be completed at baseline, post-treatment and at 3-month follow-up. Within each session, VAS regarding current ED experiences (urge to restrict, urge to binge, urge to purge, urge to exercise, feeling full, feeling fat, urge to eat, feeling low, level of stress, level of anxiety, level of tension, and current hunger) will be completed pre- and post-real/sham rTMS. The FoFM and the EDE-QS will additionally be completed weekly during treatment.
2. Other symptomatology will be measured by questionnaires including the DASS-21, Positive and Negative Affect Schedule (PANAS) [81], Profile of Mood States (POMS) [82] and Intolerance of Uncertainty Scale (IUS) [83] at baseline, post-treatment and 3-month follow-up. The DASS-21 will additionally be completed weekly during treatment.
3. Offline neuropsychological tasks assessing: i) inhibitory control as assessed by a proactive inhibition task [84] will be completed at baseline, post-treatment and 3-month follow-up, while ii) attentional bias to food using the visual probe task (VPT) [85, 86] and iii) food choice behaviour tasks (FCT) [87] will be conducted at baseline and post-treatment.
4. Questionnaires assessing: i) impulsivity/compulsivity and self-regulation, that is, Delaying Gratification Inventory (DGI) [88], Barratt Impulsiveness Scale (BIS-11) [89] and Obsessive-Compulsive Inventory (OCI-R) [90], and ii) cognitive control of emotions and self-efficacy, including the Eating Disorder Recovery Self-Efficacy Questionnaire (EDRSQ) [91], Emotion Regulation Questionnaire (ERQ) [92] and the Cognitive Flexibility Scale (CFS) [93] assessed at baseline, post-treatment and 3-month follow-up, and iii) quality of life (EuroQol Quality of Life Scale EQ-5D-5 L [94]) and illness impact (Clinical Impairment Assessment (CIA)) [95, 96] will be assessed at baseline and 3-month follow-up.

##### Intervention/service related outcomes

1. Treatment expectations, tolerability and acceptability of rTMS will be assessed by VAS and thematic analysis of semi-structured interviews.
2. Service utilisation of treatments and services other than rTMS will be assessed with a self-report version of the Clinical Service Receipt Inventory (CSRI) [97].

##### Neuroimaging outcomes

Magnetic resonance imaging (MRI) measures at baseline and post-treatment include:

1. Structural MRI for neuronavigation of the rTMS coil and for assessing whole-brain structural changes after rTMS using T1 weighted images acquired through a magnetisation-prepared 180 degree radio-frequency pulse and rapid gradient-echo (MPRAGE) sequence (voxel size: 1.1 × 1.1 × 1.2 mm, TR: 7.312 ms, TE: 3.016 ms, number of slices: 196, slice thickness: 1.2 mm, slice gap: 1.2 mm, FOV: 11, flip angle: 11°, matrix: 256 × 256 mm);
2. fMRI involving paradigms assessing inhibitory motor control in a Stop Signal Task (SST) [98] and motivation control, that is, temporal foresight and reward-related decision making in a temporal discounting (TD) task [99, 100]), using an echo planar imaging (EPI) sequence for both tasks (TR: 2,000 ms, TE: 30 ms, number of slices: 40, slice thickness: 3 mm, slice gap: 3.3 mm, number of volumes: SSRT = 193, TD = 363, FOV: 240, flip angle: 75°, matrix: 64 × 64 mm);
3. Resting state fMRI to study neural networks at rest (TR: 2,500 ms, TE: 28 ms, number of slices: 32, slice thickness: 3 mm, slice gap: 4 mm, FOV: 240, flip angle: 80°, matrix: 64 × 64 mm);
4. ASL to obtain a quantitative measure of cerebral blood flow at rest (TR: 5,135 ms, TE: 11.088 ms, number of slices: 56, slice thickness: 3 mm, slice gap: 3 mm, FOV: 240, flip angle: 111°, matrix: 512 × 8 mm).

#### Blinding

Participants and the researchers conducting assessments and delivering the rTMS will be blinded to treatment allocation throughout; that is, the study will be conducted in a triple-blind fashion. To assess whether allocation concealment has been successful, participants and the researcher will be asked to guess the treatment allocation at the end of the rTMS treatment and to indicate how certain they are of this guess. Participants (but not rTMS therapists in order for them to remain blinded throughout the study) will be debriefed and unblinded to group allocation upon completion of the 3 month follow-up. At that point participants in the sham condition will be offered real rTMS treatment following the same protocol as described above.

### Analyses

#### Feasibility

The decision as to whether to progress the study to a future large-scale RCT will be based on a number of criteria. These include the number of patients we are able to recruit, the proportion of patients retained in the study, the proportion of patients completing the real rTMS/sham intervention, the acceptability of real rTMS/sham intervention and the effect sizes of treatment outcomes. At the end of the study, these factors will be used by the study team to decide the case for progressing to a substantive RCT.

#### Clinical outcomes

Analyses will use the intention-to-treat principle. To determine quality, completeness and variability of the outcome measures, descriptive statistical analyses and graphical methods will be used. The size of the treatment effect on each outcome measure (BMI, questionnaires, neuropsychological processes) will be the difference in outcome data between those in the two treatment conditions at post-treatment and follow-up. Group differences will be estimated using linear mixed effects regression models, controlling for the baseline level of the outcome. The aim of the analysis is to establish a suitably precise effect size for the primary outcome in a future large-scale RCT at the post-treatment assessment. Additionally, multiple regression models will permit exploration of the relationship between changes in the varying neuroimaging measures and symptom improvement on questionnaire and task-based outcomes.

#### Service utilisation data

A cost-consequences approach (CCA) [101] will be used which presents the mean values of each cost category (treatment, other services use) and outcomes in each group together with appropriate measures of central tendency. A CCA does not formally analyse cost-effectiveness. Instead, it provides descriptive information that can be used to generate economic hypotheses for future definitive studies of cost-effectiveness.

#### Qualitative data

Interviews will be conducted with a minimum of ten participants from each of the groups, recruited according to key characteristics (such as previous treatment history), until theoretical saturation is reached. Interviews will explore initial expectations of the intervention, perceived strengths and weaknesses, and suggestions for improvements. Interviews will be recorded, transcribed and analysed using thematic analysis [102].

#### Neuroimaging analysis

##### Structural MRI

Voxel-based morphometry (VBM) analyses will be conducted to evaluate any morphological changes resulting from the series of rTMS.

##### ASL

ASL data will be evaluated in a similar manner to the structural MRI. Following pre-processing, second-level models (such as ANOVAs) will be used to test for an effect of treatment.

##### Resting state MRI

Hypothesis-led seed-based connectivity (DLPFC seed) analyses will be conducted before and after treatment to explore the effect of rTMS on cortico-cortical and thalamo-cortical connectivity. Note, scans will not immediately follow rTMS and therefore will not be affected by acute effects of rTMS.

##### fMRI

Mass univariate analysis will be implemented within SPM12 under the standard generalised linear model (GLM). Contrasts of parameter estimates for separate conditions will provide context-specific maps of neural activity for each participant/session. These will include neural activation to delayed compared to immediate choices in the TD task. In the SST, the main contrasts of interest will include (a) failed stop versus successful stop trials; (b) failed stop versus successful go trials; (c) successful stop versus successful go trials.

##### Common elements

For all modalities, separate images (connectivity maps, task-related activation, structural parameters such as grey matter volume) will be taken forward into a second-level random effects analysis, implemented within SPM12. This will necessitate a factorial ANOVA model, with group (real versus sham) and time (pre versus post), with a significant interaction the primary contrast of interest, as with the clinical and questionnaire data. Significance will be defined as correction for multiple comparisons (*p*FWE < 0.05) at a given height threshold of Z-score of 2.3.

## Discussion

Treatment outcomes for psychotherapies are modest for adults with AN, and there is currently no gold-standard treatment [103]. Given the paucity of effective treatments for adult AN, specifically SEED-AN, new treatments are needed. In light of this, and with the growing understanding of the neural underpinnings of AN, research into novel non-invasive brain-directed therapeutic approaches is warranted [28, 45, 46]. This paper has outlined the protocol for a feasibility trial that will inform future studies (for example, provide effect sizes for a large RCT) and add to the evidence for brain-directed interventions for AN [57, 58, 104], McClelland et al., unpublished observations. Strengths of the study include the use of an individualised (neuronavigated) neuromodulation technique. Moreover, the protocol is designed to probe disease mechanisms (that is, with the inclusion of multiple structural and functional neuroimaging measures) and will provide important insight into the neural correlates of the therapeutic effect. Finally, the protocol adheres to guidance on the optimal conduct of neuromodulation trials [105–107].

Several potential practical and operational issues may pose challenges to the timely completion of this study, particularly with regards to recruitment and attrition. Patients with AN are often ambivalent about treatment and this is reflected in poor take-up rates of certain treatments (for example, medications leading to weight gain) and high drop-out rates [108]. Whilst rTMS appears promising in AN, it is unclear what the take-up, attendance, and retention rates will be if this is offered to SEED-AN patients. Although participants who took part in our previous single-session rTMS trials [McClelland et al., unpublished observations] showed interest in having rTMS therapeutically, adherence and completion of the treatment may prove challenging, for example, if the participant believes they are receiving sham, if the treatment is too uncomfortable or if the research is too cumbersome.

Although we are using the most up-to-date equipment with an improved sham coil that elicits a more realistic TMS-like sensation, it is not clear whether participants, especially those who may have previous experience of rTMS in other research studies, will be able to distinguish between the real and sham treatment and what impact this will have on attrition rates. However, in our previous RCT of a single rTMS session in people with AN there was no difference between groups in the ability to correctly guess stimulation type [McClelland et al., unpublished observations].

It will be important to ascertain patients’ willingness to undergo random allocation to real or sham rTMS. This is not trivial, as rTMS is a relatively demanding treatment, requiring daily attendance for an extended period. However, this type of RCT is considered the gold-standard method of evaluating the clinical efficacy of rTMS treatment in other disorders, such as depression [50]. Providing participants who were randomised to receive sham rTMS with the opportunity to receive real rTMS after they have completed the study is thought to be critical in encouraging recruitment and participant retention. Addressing these challenges will be particularly useful in informing the development of future large-scale RCT of rTMS in EDs.

In summary, research into novel treatments for SEED-AN is essential. rTMS is a promising neuromodulatory technique that has shown preliminary benefit in AN, including individuals with SEED-AN. This innovative feasibility RCT will be the first to systematically assess the acceptability, efficacy and neural correlates of this promising treatment in comparison to an active control condition. This will provide a foundation for the development of future large-scale RCTs and, if the results are positive, will provide support for the implementation of this as a treatment adjunct in clinical practice.

## Trial status

Participant recruitment and data collection for this study began in August 2015.

## Additional file

Additional file 1:
**TIARA study schedule of enrolment, interventions and assessments.** This table presents the time schedule of enrolment, interventions and assessments, consistent with the figure provided in the SPIRIT Statement (2013) recommendations for reporting protocols. (DOCX 20 kb)

## Abbreviations

AN: Anorexia Nervosa
ASL: arterial spin labelling
BIS: Barratt Impulsiveness Scale
BN: Bulimia Nervosa
CFS: Cognitive Flexibility Scale
CIA: Clinical Impairment Assessment
CSRI: Clinical Service Receipt Inventory
DASS-21: 21 item Depression, Anxiety and Stress Scale
DGI: Delayed Gratification Inventory
EDs: Eating Disorders
EDDS: Eating Disorder Diagnostic Scale
EDEQ: Eating Disorder Examination Questionnaire
EDRSQ: Eating Disorder Recovery Self-Efficacy Questionnaire
EQ-5D-5 L: EuroQol Quality of Life Scale
ERQ: Emotion Regulation Questionnaire
FCT: Food Choice Task
fMRI: functional magnetic resonance imaging
FoFM: Fear of Food Measure
GNG: Go/No-Go Task
IUS: Intolerance of Uncertainty Scale
MRI: magnetic resonance imaging
OCI: Obsessive-Compulsive Inventory
PANAS: Positive and Negative Affect Schedule
POMS: Profile of Mood States
RCT: randomised controlled trial
rTMS: repetitive Transcranial Magnetic Stimulation
SCID: Structured Clinical Interview for DSM Disorders
SEED-AN: severe and enduring Anorexia Nervosa
SS: Self-Starvation Scale
SST: Stop Signal Task
TAU: treatment as usual
TD: temporal discounting
VAS: visual analogue scale
VPT: Visual Probe Task

## Footnotes

1 Some authors have reserved this term for people with an illness duration > 7 to 10 years [23, 24].
