# Supplementary material for: Clinical outcomes and neural correlates of 20 sessions of repetitive transcranial magnetic stimulation in severe and enduring anorexia nervosa (the TIARA study): study protocol for a randomised controlled feasibility trial
Source: Trials. 2015 Dec 3;16:548. doi: 10.1186/s13063-015-1069-3 (PMC4668644; doi:10.1186/s13063-015-1069-3)
Supplement: Additional file 1: — TIARA study schedule of enrolment, interventions and assessments. This table presents the time schedule of enrolment, interventions and assessments, consistent with the figure provided in the SPIRIT Statement (2013) recommendations for reporting protocols. (DOCX 20 kb) [file 13063_2015_1069_MOESM1_ESM.docx]

|  | **STUDY PERIOD** | | | | | | | | | | | | | | | | |
| --- | --- | --- | --- | --- | --- | --- | --- | --- | --- | --- | --- | --- | --- | --- | --- | --- | --- |
|  | **Treatment (real rTMS) group and control (sham rTMS) group** | | | | | | | | | |  | **Control group only (optional)** | | | | | |
|  | **Enrolment** | **Pre-allocation** | **Allocation** | **Treatment** | | | | **Post-treatment** | | |  | **Treatment** | | | | **Post-treatment** | |
| **TIMEPOINT** | ***0*** | **Baseline assessment (t1)** | **0** | ***Week 1*** | ***Week 2*** | ***Week 3*** | ***Week 4*** | ***Follow up (t2)*** | ***Qualitative interview*** | ***3 month follow up (t3)*** |  | ***Week 1*** | ***Week 2*** | ***Week 3*** | ***Week 4*** | ***Follow up (t4)*** | ***3-month follow up (t5)*** |
| **ENROLMENT:** |  |  |  |  |  |  |  |  |  |  |  |  |  |  |  |  |  |
| **Eligibility screen** | X |  |  |  |  |  |  |  |  |  |  |  |  |  |  |  |  |
| **Informed consent** | X |  |  |  |  |  |  |  |  |  |  |  |  |  |  |  |  |
| **Allocation** |  |  | X |  |  |  |  |  |  |  |  |  |  |  |  |  |  |
|  |  |  |  |  |  |  |  |  |  |  |  |  |  |  |  |  |  |
| **INTERVENTIONS:** |  |  |  |  |  |  |  |  |  |  |  |  |  |  |  |  |  |
| ***Real rTMS*** |  |  |  | X | X | X | X |  |  |  |  | X | X | X | X |  |  |
| ***Sham rTMS*** |  |  |  | X | X | X | X |  |  |  |  |  |  |  |  |  |  |
|  |  |  |  |  |  |  |  |  |  |  |  |  |  |  |  |  |  |
| **ASSESSMENTS:** |  |  |  |  |  |  |  |  |  |  |  |  |  |  |  |  |  |
| ***Weight*** | X | X |  | X | X | X | X | X |  | X |  | X | X | X | X | X | X |
| ***Questionnaires*** |  | X |  |  |  |  |  | X |  | X |  |  |  |  |  | X | X |
| ***Computer Tasks*** |  | X |  |  |  |  |  | X |  | X |  |  |  |  |  | X | X |
| ***MRI*** |  | X |  |  |  |  |  | X |  |  |  |  |  |  |  |  |  |
| ***Monitoring blood samples*** | X | X |  |  | X |  |  |  |  |  |  |  | X |  |  |  |  |
| ***Within-session VAS scales*** |  |  |  | X | X | X | X |  |  |  |  | X | X | X | X |  |  |
| ***Qualitative interview*** |  |  |  |  |  |  |  |  | X |  |  |  |  |  |  |  |  |
|  |  |  |  |  |  |  |  |  |  |  |  |  |  |  |  |  |  |
